# Brine Solution with Hydrocolloids Used to Enhance the Properties of Sterilized Meat

**DOI:** 10.17113/ftb.58.02.20.6336

**Published:** 2020-06

**Authors:** Vinicius Jose Bolognesi, Michele Rigon Spier, Carlos Eduardo Rocha Garcia

**Affiliations:** 1 Pharmacy Department, Pharmaceutical Sciences Postgraduate Programme, Federal University of Parana, Lothario Meissner St. 632, 80210-170 Curitiba, Paraná, Brazil; 2 Chemical Engineering Department, Federal University of Parana, Francisco H. dos Santos St., 81530-900, Curitiba, Paraná, Brazil

**Keywords:** collagen in meat, lipid oxidation during shelf life, thermal inactivation of ready-to-eat foods, water retention

## Abstract

**Research background:**

Retort processing is one of the most widely used methods of thermal inactivation that provides convenient, ready-to-eat foods. Although this technology remains widespread, it can be revamped through processing of novel ingredients such as gums. This article aims to investigate the effect of the hydrocolloids collagen, soy protein isolate, carrageenan and modified starch with different salt mass fractions on the retorted meat products.

**Experimental approach:**

Firstly, solutions of the added hydrocolloids of different salt mass fractions in order to stimulate the salting-in effect were studied. Lipid oxidation, syneresis and water activity were analysed during shelf life to find the best overall treatments. Lastly, sensory and texture analyses were then performed to assess the impact of the added hydrocolloids.

**Results and conclusions:**

Yield, cooking loss and water-holding capacity had better results when higher salt mass fractions with hydrocolloids were used. The physicochemical results distinguished collagen from the other tested hydrocolloids. Syneresis remained in similar ranges regardless of the treatment. No difference was observed in water activity either. However, sterilization, vacuum sealing and the addition of a hydrocolloid contributed to low oxidation levels in all treatments. Lastly, sensory, texture and shear force analyses confirmed that the products with collagen were harder and firmer than the control samples, which explains the preference of control samples by the panellists. Nevertheless, assessors did not perceive the presence of collagen.

**Novelty and scientific contribution:**

Physicochemical and sensory characteristics of the retorted meat can be considerably improvedwhen brine and hydrocolloids are combined with the retort technology.

## INTRODUCTION

Sterilization is a widespread technology that can offer meat products with desirable physicochemical and organoleptic parameters (*1*), the most important being the ability to provide a safe, convenient and ready-to-eat food products (*2*). However, sterilization under high temperatures affects meat proteins, causing unfolding which leads to a lower binding capacity with water, influencing the texture and flavour, and resulting in yield loss (*2*-*4*). Even though several meat products have been successfully developed by retorting, such as meatballs (*5*) and chicken porridge (*6*) among others, there is a lack of studies showing the benefit of the use of hydrocolloids with this kind of processing.

Biopolymers have the ability to retain water through their hydrophilic groups and are temperature-dependent. The heating and cooling stages of sterilization instil gelatinization on hydrocolloids, improving, for example, the formation of a resistant starch, viscosity (*7*) and water retention (*8*, *9*). Water can be found in three different forms in the polysaccharide-water systems, *i.e.* non-freezing, freezing bound and free water. Between the two kinds of bound water, the amount of non-freezing water depends on the chemical structure of the polysaccharide matrix. In the case of water-insoluble polysaccharides, the number of hydroxyl groups located in the amorphous region determines the amount of non-freezing water. In contrast, the amount of freezing bound water depends on the higher-order structure of the molecular chain (*9*).

In order to obtain optimal yield and ensure safety of the retorted meat products, the of the interaction between hydrocolloids and meat under the processing conditions must be investigated (*8*, *10*). Therefore, before such a system can be developed and commercialized, it is important to know the behaviour of the additives in meat products (*1*).

Hence, this work aims to investigate the influence of the individual use of hydrocolloids collagen, soy protein isolate, carrageenan, and modified starch, with different sodium chloride mass fractions on yield, cooking (by retorting) loss, water holding capacity and, subsequently, assess the shelf life by evaluating the parameter of oxidation stability, water activity and syneresis on the sterilized beef.

## MATERIALS AND METHODS

### Materials

Beef muscle (*vastus lateralis*) was purchased from a local market in Curitiba, Brazil, and stored under refrigeration ((4±2) °C) until further processing. The hydrocolloids used were: carrageenan, soy protein isolate, collagen (all Global food, Sao Paulo, Brazil) and modified starch (Ingredion, Balsa Nova, Brazil). The brine solution of hydrocolloids consisted of sodium chloride (Reagen, Colombo, Brazil) and distilled water. Ether petroleum was purchased from Vetec (Duque de Caxias, Brasil). Thiobarbituric acid was obtained from Sigma-Aldrich, Merck (St. Louis, MO, USA). All reagents were of analytical grade.

### Proximate chemical composition

Samples of the muscle *vastus lateralis* were used for the analysis of chemical composition. Moisture content was determined according to AOAC official method 950.46 (*11*). Approximately 10 g of the sample were weighed in a moisture dish and the mass was recorded. The dish was then placed in a hot air oven (model 420-2D; Nova Etica, Sao Paulo, Brazil) at (105±2) °C until a constant mass was recorded. The samples were weighed again and the moisture content was then calculated from the following formula:


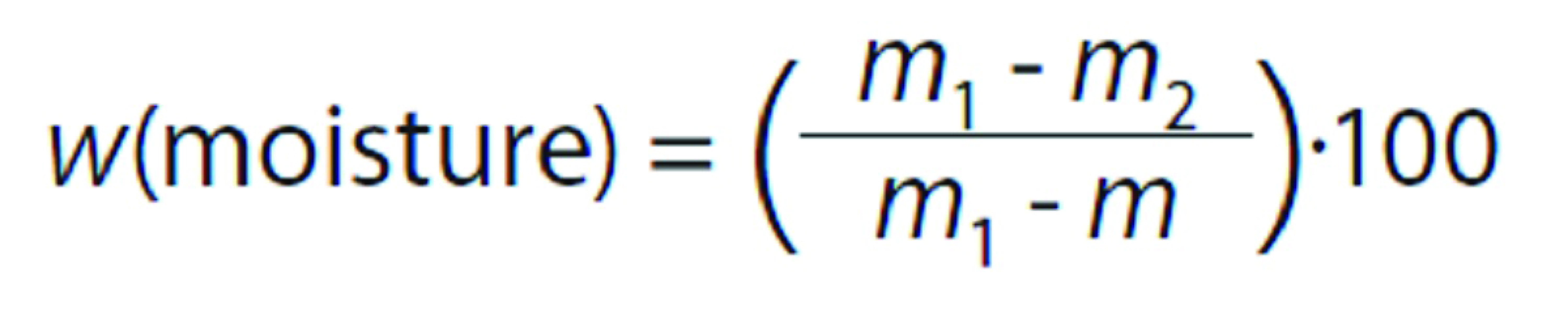


where *m* is the mass of the empty dish (g), *m*_1_ is the initial mass of the dish containing the sample (g), and *m*_2_ is the final mass of the dish with the sample after drying (g).

The pH value was measured with a meat pH meter (model mCA-150; MS Tecnopon, Sao Paulo, Brazil) inserted into the raw meat.

The Kjeldahl method was used to determine the nitrogen content of the meat samples according to the following equation:


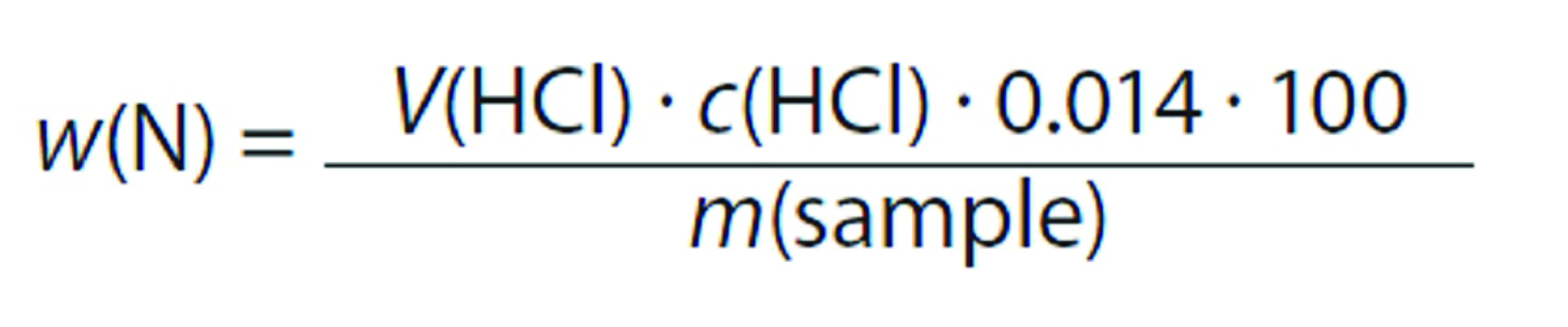


Protein content was determined by multiplying the quantity of nitrogen by nitrogen-to-protein conversion factor 6.25, as described in AOAC official method 928.08 (*12*). The fat mass fraction was determined by an ether petroleum extraction. Samples (5 g) were inserted into a fat extraction flask that had been previously weighed, it was connected to the Soxhlet apparatus (model SL 145/6; Solab, Sao Paulo, Brazil), and then subjected to a continuous extraction with ether petroleum for 6 h. The fat extraction flask was then removed from the extractor and allowed to dry for 2 h at 40 °C in a hot air oven (model 3/495; De Leo, Porto Alegre, Brazil) until no trace of ether petroleum remained. The fat content was determined from the obtained dry mass of moisture essay, according to the AOAC official method 960.39 (*13*).

Mineral residue content (*14*) was determined by heating the samples in a muffle furnace (model Q318 24; Quimis, Diadema, Brazil) at 550 °C for 24 h. Mineral residue was also gathered during the retorted control treatments with NaCl 2.5 and 5.0%.

### Brine solution of hydrocolloids prepared for retorting

Sterilized beef was treated with a fixed 1.0% hydrocolloid and a brine volume fraction of 40%. These set values were obtained from previous experiments of a 3^2^ factorial design to estimate the optimal hydrocolloid mass fraction and water volume for injection in beef (data not shown).

The brine solution containing hydrocolloids was prepared as follows: mass fraction of 2.5 or 5.0% NaCl with 1.0% hydrocolloid (except control, which did not contain a hydrocolloid) were weighted and solubilized with 40% of distilled water. After mixing, the solutions were stirred (model 6795-400D; Corning, Corning, NY, USA) for 10 min at 25 °C and 1200 rpm.

The beef samples included treatments with carrageenan, soy protein isolate, collagen, modified starch and control. They were portioned in cubes (3 cm×3 cm×3 cm) of approx. 45 g and equal quantities separated according to each treatment. In addition, NaCl at mass fractions of 2.5 or 5.0% was added to all treatments to activate the salting-in effect.

Finally, the beef cubes were injected with the solutions and remained immersed in the respective solution for 12 h at 4 °C. Then, the beef cubes were drained and vacuum-packed for sterilization, as described below.

### Thermal process evaluation

The sterilization was performed in a water cascading retort (Ardode, Araquari, Brazil) operating at 121 ºC and 0.150 MPa, conditions similar to the commercially retorted meat products. After processing the pouches to the required *F*_0_ value, they were cooled rapidly by pumping and recirculating water into the chamber until the core temperature of the product reached 30 °C.

For heat penetration studies, four pouches had the type T thermocouples (Exacta, Sao Paulo, Brazil) inserted and sealed with a heat-resistant silicone, and they were fixed in different positions in the chamber to determine the lowest heating point of the meat cubes. Then, the temperature at the slowest heating point of the retort pouches was monitored. The thermocouple tips were inserted into the beef cubes positioned in the geometric centre of the pouch. Temperature outputs of the thermocouples were recorded every 60 s, and sterility was expressed as an *F*_0_ value, using the Simpson rule (*15*), calculated with the following equation:


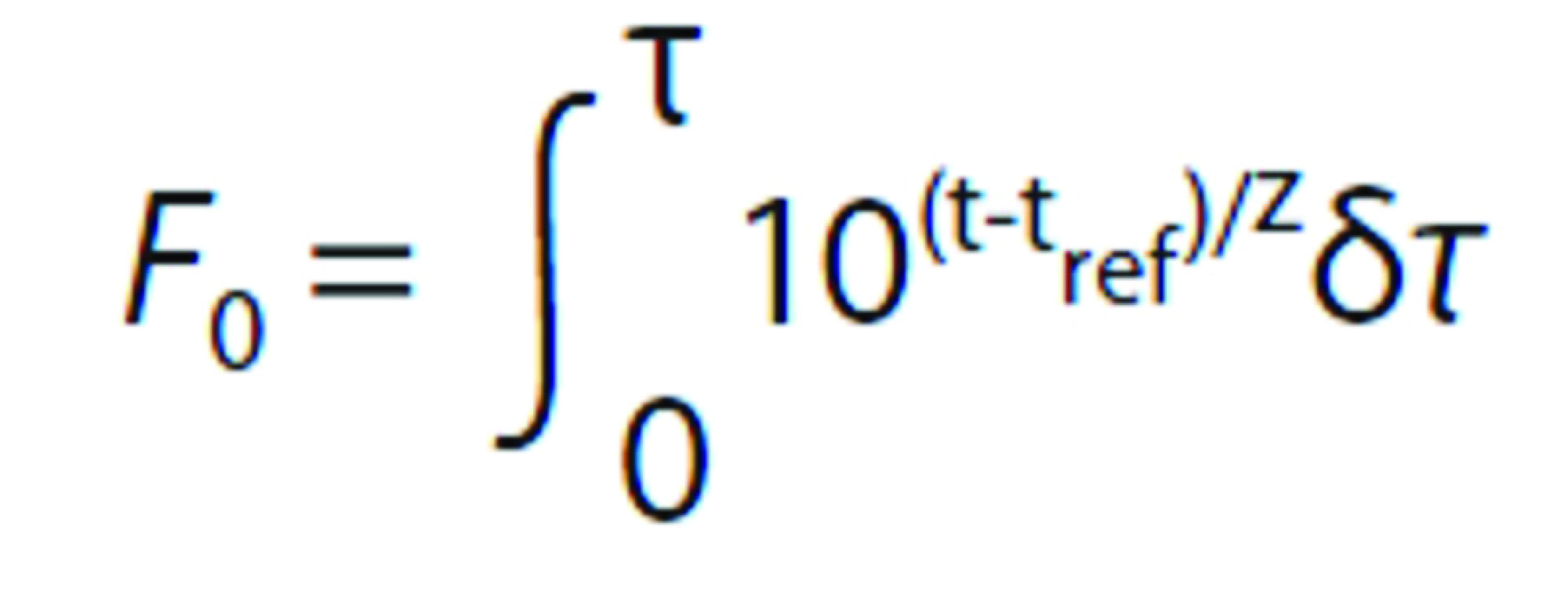


where *F*_0_ is the calculated lethality value (min), *t* is the temperature of the thermocouple (°C), *t*_ref_ is the reference temperature, z is the temperature coefficient for microbial destruction (°C), and *τ* is time (min).

### Measurements of moisture, yield, water holding capacity and cooking loss

Moisture, yield (*Y*/%), water-holding capacity (WHC/%), and cooking loss (CL/%) of the sterilized meat samples were analyzed before and after the sterilization. Moisture was evaluated according to AOAC (*11*) as mentioned earlier. The yield was determined by weighing the samples before and after the injection of the brine solution containing hydrocolloids, and cooking loss was calculated by weighing the drained beef cubes after sterilization.

Loss of mass during different stages of processing was carefully monitored through yield, cooking loss and the total mass loss. All losses were calculated as a percentage of mass taken prior to each processing stage.

Water-holding capacity measurement followed the procedure described by Ayadi *et al.* (*16*). About 10 g of each cube sample was centrifuged (model Z323K; Hermle, Gosheim, Germany) at  10 200×*g* for 30 min at 4 °C. The water-holding capacity was calculated as a percentage of the bound water using the following equation:


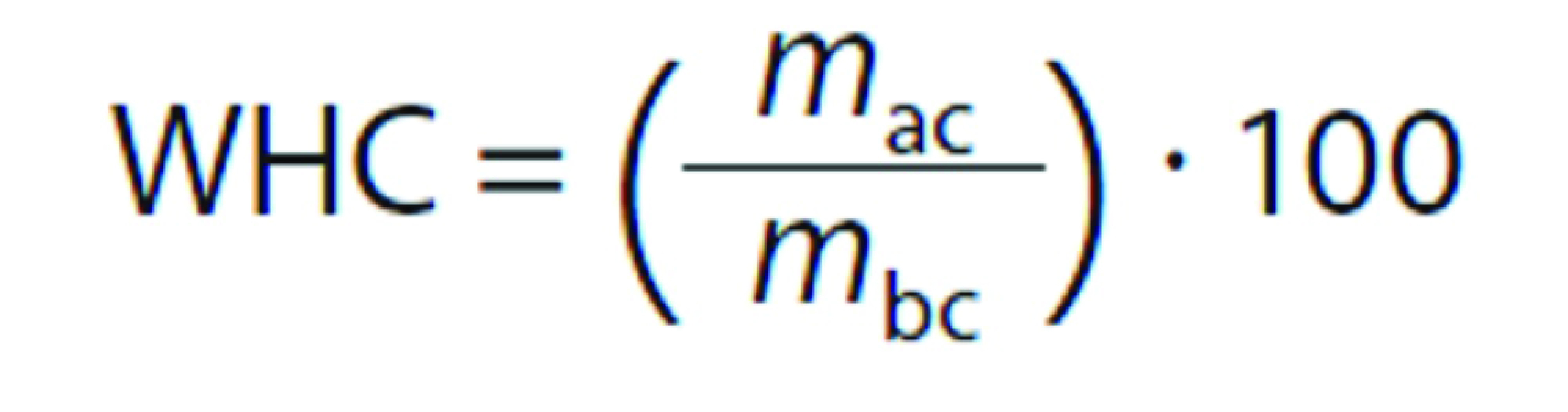


where WHC is the water-holding capacity (%), *m*_ac_ is the mass of the beef sample after centrifugation, and *m*_bc_ is the mass of the sample before centrifugation.

### The shelf life of the sterilized beef

The treatments that had the best yield, water-holding capacity and optimal cooking loss at 5.0% NaCl were tested during 180 days of storage at 25 °C in a refrigerator (model TE-391; Tecnal, Piracicaba, Brazil).

Samples were drained from the brine solutions and weighed before sterilization. In order to validate the efficiency of the sterilization in reducing contamination, sterilized samples (500 g) collected on the first day of storage were sent to an outsourced microbiology laboratory, where the following microbiology assays were performed: faecal coliforms according to Association Française de Normalisation (AFNOR) (*17*), enumeration of sulphite-reducing *Clostridium* according to ISO 15213:2003 (*18*), determination of *Salmonella* spp. and coagulase-positive *Staphylococcus* (*19*). Syneresis was evaluated according to Honikel (*20*). After sterilization, the pouches were opened each storage day, the exudate was drained from the samples and before weighing them again. The difference in the mass of the drained samples before and after sterilization was calculated as follows:


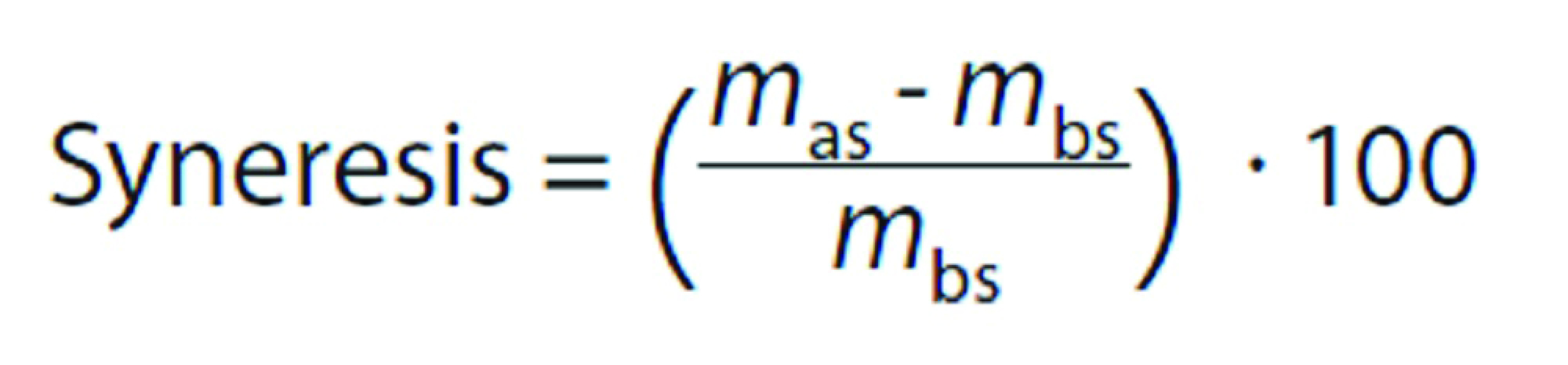


where *m*_as_ is the mass of the beef sample after sterilization, and *m_bs_* is the mass of the sample before sterilization.

Water activity (*a*_w_) was determined with a water activity meter model AquaLab CX-2 (Decagon Devices Inc., METTER Group, Pullman, WA, USA), using a controlled temperature of (25±1) ºC.

The extent of lipid oxidation was assessed by analysis of the 2-thiobarbituric acid reactive substances (TBARS) using the distillation method described by Torres *et al.* (*21*). The TBARS values were expressed in mg malondialdehyde (MDA) per kg sample. Three samples of (10±0.001) g from each treatment were measured in sextuplicate.

### Texture profile analysis and shear force

A double compression cycle test was performed (texture analyzer model CT3; AMETEK Brookfield, Middleborough, MA, USA) up to 50% compression of the original portion height with a nylon cylinder probe of 2 cm in diameter. Time of 1 s was allowed to elapse between the two compression cycles. Force–time deformation curves were obtained with a 25 kg load cell applied at a cross-head speed of 3 mm/s. The following parameters were quantified: hardness, cohesiveness, springiness, and chewiness.

The Warner–Bratzler shear force was measured using the method described by Wheeler *et al.* (*22*). Six cores of at least 2.5 cm length and 1.27 cm in diameter were excised from the cooked samples. The direction of muscle fibres was parallel to the longitudinal direction of the core. The cores were tested on a texture analyser (model CT3; AMETEK Brookfield) that had a 1 mm thick cutting V-shaped blade and speed of 3 mm/s when cutting through the core. Shear force was recorded as peak force (N).

### Sensory analysis

Sensory analysis was performed according to the method described by Nicola (*23*). The samples were heated in a water bath at 100 °C until the temperature in the centre reached 70 °C. For each sensory test, the meat samples were put on white, opaque plates and coded with three-digit numbers chosen randomly. All sessions were performed in a sensory analysis laboratory equipped with individual testing booths held at a constant temperature (20 °C), positive airflow removed any odours from the testing area and controlled lighting to neutralize any possible differences in colour or appearance of the meat. Saline water (0.9% NaCl solution at room temperature) was provided as a palate cleanser for rinsing the mouth and cleaning the tongue before testing each sample. To minimise the effect of tasting order, an equal number of plates with opposite sample order was prepared.

A paired preference test was conducted to assess if one of the two meat samples had more acceptable sensory characteristics, expressed as a generic preference. Assessors (*N*=74) were asked to taste the two samples starting from the left side of the plate. After tasting, they were asked to express their meat taste preference. Since a forced-choice procedure was adopted, a sample was chosen even if the selection by the assessor was random. Each person made one or two independent tastings, with a different sample order if two tastings were made.

A triangle test was performed (*N*=74) where judges were asked to detect any change in the taste and mouthfeel of the treated sample as compared to the changes in the control sample. Assessors were asked to smell and taste one different and two identical samples of meat in the same session and instructed to indicate the odd sample.

### Statistical analysis

Analyses of the proximate composition, physicochemical characteristics, texture and shear force were performed in quadruplicate. Lipid oxidation was measured in sextuplicate. The results were reported as mean value±standard deviation. The differences between mean values were determined using analysis of variance (ANOVA) with Tukey’s test (p<0.05) and their statistical significance with the StatSoft STATISTICA v. 12.5.1 (*24*) software.

## RESULTS AND DISCUSSION

### Proximate composition and pH

The findings of the proximate composition and other physicochemical parameters shown in Table 1 were found to be in line with other studies about *vastus lateralis* used as raw meat in meat products (*4*).

**Table 1:** Compositional and physicochemical parameters of raw *vastus lateralis* meat

Parameter		Value
*w*(moisture)/%		73.0±1.1
*w*(fat)/%		5.7±0.5
*w*(protein)/%		21.6±0.8
*w*(mineral)_residual_/%	Raw meat	1.04±0.04
	*w*(NaCl)_control_=2.5%	3.1±0.2
	*w*(NaCl)_control_=5.0%	1.5±0.3
pH		5.80±0.07)
Data represent mean value±S.D., *N*=4		

An increase in the sodium salts agitates the surface free energy of the protein-solvent interactions (*25*), whereas salt solubilizes the myofibrillar proteins in meat, which allows the proteins to increase hydration and the water-binding capacity. A review by Ruusunen and Puolanne (*26*) suggested a hypothesis to explain the role of NaCl in water-binding capacity in meat, chloride ions tend to penetrate the myofilaments causing them to swell (*27*), while Offer and Knight (*28*) and Offer and Trinick (*29*) claimed that the Na ions form an ion ’cloud’ around the filaments.

As hypothesized, an increase of the mineral residue content was observed as the NaCl concentration increased, whereas the mineral content of the control samples with 2.5 and 5.0% NaCl increased almost twofold, from (1.5±0.3) to (3.1±0.2) % (Table 1). The purpose of adding salt was to promote its functional effect on the meat proteins, which would act in favour of the hydration of the beef proteins. However, since there is a limit to the salt mass fraction for the occurrence of these effects, two mass fractions were tested. The increase in mineral content reflected the water retention properties by meat samples, as discussed in the subsequent sections.

### Thermal process evaluation of sterilized meat samples

The beef samples in the present study were sterilized for 30-35 min, resulting in an *F*_0_ of 12.98 min (data not shown), in agreement with the *F*_0_ range for meat products, which is 8-20 min according to Footitt *et al.* (*30*).

Previous experiments have demonstrated that at first, the greater yield was reached as more brine volumes were used, but cooking loss was greater and water-holding capacity decreased. In addition, with more exudate, more moisture and nutrients are available to microorganisms, as observed in the post freeze/thaw process (*31*). Thus, the optimal conditions for sterilization process were 40% brine volume fraction and 1.0% hydrocolloid (data not shown).

Addition of brine to beef samples resulted in an increase in the yield, as observed for all treatments with both 2.5 (Table 2) and 5.0% NaCl (Table 3). The hydrocolloid treatments gave at least 1% higher yield of the mass gain than control. Overall, the yield of meat was higher at 5.0% salt regardless of the treatment. Nonetheless, collagen had a significantly higher yield than control at 2.5% salt, whereas at 5.0% NaCl collagen or soy protein isolate had greater yield than control (p<0.05).

**Table 2:** Physicochemical parameters of beef (*vastus lateralis*) cubes with added 1.0% hydrocolloid and 2.5% sodium chloride

Hydrocolloid	*Y*/%	*w*(CL)/%	*Y*_total_/%	*w*(moisture)/%		WHC/%	
Before retort	After retort	Before retort	After retort
Carrageenan	(8.3±0.9)^a^	(42.3±1.6)^a^	-34.0	(77.0±1.5)^a, b^	(65.3±1.0)^ab^	(53.3±0.2)^a^	(82.0±0.3)^a^
Soy protein isolate	(7.7±1.7)^a^	(44.6±0.7)^ab^	-36.8	(75.9±1.0)^a^	(64.1±0.7)^ab^	(66.1±2.0)^b^	(86.9±2.4)^b^
Collagen	(20.0±5.1)^b^	(47.0±2.9)^b^	-27.0	(78.6±1.0)^b^	(65.3±1.5)^ab^	(73.1±4.8)^c^	(82.7±1.6)^a^
Modified starch	(9.5±1.8)^a^	(43.4±1.4)^a^	-33.9	(77.6±0.9)^ab^	(66.4±1.8)^a^	(68.6±3.1)^bc^	(82.3±1.1)^a^
Control	(7.3±1.0)^a^	(44.8±1.9)^ab^	-37.6	(78.3±0.8)^ab^	(63.3±1.0)^b^	(69.2±1.2)^bc^	(84.4±1.4)^ab^
Data represent mean value±S.D., *N*=4. Mean values in the same column with different letters in superscript are significantly different (p<0.05) according to the Tukey’s test. *Y=*Yield, CL=cooking loss, WHC=water holding capacity. *Y*_total_=total yield obtained by subtracting yield with cooking loss							

**Table 3:** Physicochemical parameters of beef (*vastus lateralis*) cubes with added 1.0% hydrocolloid and 5.0% sodium chloride

Hydrocollloid	*Y*/%	*w*(CL)/%	*Y*_total_/%	*w*(moisture)/%		WHC/%	
Before retort	After retort	Before retort	After retort
Carrageenan	(14.5±1.9)^a, b^	(43.3±3.9)^a, b^	-28.8	(70.3±1.8)^a^	(65.1±1.3)^a^	(72.5±0.7)^a^	(80.6±3.2)^a^
Soy protein isolate	(16.0±2.2)^a^	(43.1±2.4)^a, b^	-27.1	(76.3±0.7)^b^	(64.5±0.5)^a^	(70.5±2.7)^a^	(82.5±0.6)^a^
Collagen	(17.7±1.3)^a^	(43.9±2.07)^a, b^	-26.2	(74.5±1.4)^b^	(63.1±2.5)^a^	(71.8±4.1)^a^	(78.9±0.6)^a^
Modified starch	(13.7±3.5)^b^	(41.0±1.28)^a^	-27.3	(74.5±2.3)^ab^	(65.3±0.8)^a^	(74.6±6.9)^b^	(83.7±2.5)^a^
Control	(11.6±1.3)^b^	(44.8±2.78)^b^	-33.2	(74.8±2.3)^b^	(62.9±2.6)^a^	(68.5±5.7)^a^	(80.5±4.7)^a^
Data represent mean value±S.D., *N*=4. Mean values in the same column with different letters in superscript are significantly different (p<0.05) according to the Tukey’s test. *Y=*Yield, CL=cooking loss, WHC=water holding capacity. *Y*_total_=total yield obtained by subtracting yield with cooking loss							

Cooking loss was the lowest in the samples treated with carrageenan and modified starch at 2.5% salt (p<0.05) (Table 2), while at 5.0% salt, no significant differences were observed among the treatments (Table 3). As the yield was higher in meat samples treated with hydrocolloids, regardless of the salt mass fraction, similar cooking loss as in the control indicated that more water remained in the matrix even after the thermal treatment.

Hydrocolloids, such as the water-soluble polysaccharides, change their molecular conformation in aqueous media as a function of temperature (*1*, *8*, *9*). The typical gel-forming hydrocolloids (*e.g.* agar, carrageenan, gellan and gelatin) undergo a molecular rearrangement (*i.e*. from random coil to a helicoidal state) as a prerequisite to gel formation. However, the created gel is under the influence of the kinetic control and the rate at which gelation is induced with the increase of temperature or the salt mass fraction (in systems where gelation is ion mediated) (*32*).

The yield of the samples treated with hydrocolloids was higher than of the control (Table 3). Hsu and Chung (*33*) found a higher yield in cooked emulsified meatballs with 20% water and κ-carrageenan and salt mass fractions of 1.6-2.0 and 1.8-2.2% respectively. Myofibrillar proteins have an essential water-binding role in meat products through, for example, the heat gelation process (*32*), and are influenced by factors such as salt mass fraction (ionic strength) and the presence of non-protein polymer ingredients (*26*, *34*).

Total muscle protein comprises about 60% of the salt-soluble proteins (*10*). Chloride and sodium ions have a strong bond with myofibrils, shifting the net charges of the proteins that exert effects on hydration, such as salting in or salting out (*26*). The salting in effect, in which myofibrillar salt-soluble protein chains bind water molecules was observed by Offer *et al.* (*28*), who found that as the salt content of a salting solution increased above the physiological ionic strength of meat, there was a progressive increase in the amount of water-holding capacity.

Besides improvements in the water-holding capacity, the effect of salt ions (*27*) was at maximum level when ionic strength of the solution was 1.0 M (5.8% NaCl) (*29*). The brine solutions used in this work were roughly 0.5 M (2.5% NaCl) and 1 M (5.0% NaCl), which provided an increase in the mineral content from 1.04% (raw meat) to 3.1% (5.0% NaCl) and had a synergistic effect with hydrocolloids on moisture retention (Table 1).

The salting in effect was evident in the yield of the control treatment, which increased from 7.3% when using 2.5% NaCl (Table 2) to 11.6% when using 5.0% NaCl (Table 3). Much higher yield was obtained in beef samples treated with collagen, which was unexpected since gelatinization and loss of collagen have been reported above 80 °C (*3*).

As seen in Table 3, part of the mass gain in treatments with hydrocolloids was maintained after the thermal treatment. The water-holding capacity values, which remained the same (p>0.05) compared to those of the control, displayed the mechanism by which hydrocolloids entrapped water inside the samples. The water that was incorporated in the matrix, which resulted in a higher yield, not only remained but was also deeply retained in the matrix. Ayadi *et al.* (*16*) showed that increasing the carrageenan mass fraction from 0 to 1.5% caused an increase in the water-holding capacity of about 1% in sausages. Even though higher yield was observed for all treatments (Table 3), there was no increase in *a*_w_. This can be explained by the net charge difference promoted by the salting in effect (*25*). Since the structural proteins in meat cannot move, electrical forces pull the sodium ions very close to the filament surfaces, creating an uneven distribution of ions in the water phase. This establishes an osmosis-like force within the filament lattice, which in turn pulls water molecules into the system (*26*).

Partial loss of brine is expected before thermal processing, resulting in the loss of the injection volume retained within the matrix. McDonald *et al.* (*35*) found that all brine phosphate injections (20-45%) in beef had similar cooking losses, suggesting that not all the phosphate and salt were incorporated into the samples. Sheard and Tali (*36*) injected 10% brine solutions at in pork loin and found that salt or bicarbonate alone had higher drip losses than those in the control samples.

However, most water in the muscle is held within the myofibrils (*25*). The bound water, which exists in the vicinity of non-aqueous constituents (like proteins) is very resistant to freezing and removal by conventional heating. In addition, entrapped water may be held either by steric (space) effects and/or by attraction to the bound water (*25*).

Therefore, the water is entrapped in the meat matrix due to salt solubilization and the functional properties of hydrocolloids. A study by Gou *et al.* (*37*) showed that pork ham samples soaked in 5 kg NaCl per 100 kg H_2_O had the highest moisture content on wet mass basis.

### Shelf life of retorted beef

Treatments with collagen and modified starch at 5.0% NaCl were chosen for shelf life analysis. The samples had the best overall values in all treatmens with 5.0% salt Table 2 and Table 3). The choice of hydrocolloids was determined by total yield (Table 3). Total yield gives an overall aspect of which treatment can provide not only a higher yield, but also maintain the mass gain after the thermal treatment (*33*). Even though soy protein isolate had similar (p>0.05) values to collagen and modified starch, for industrial purposes soy proteins are limited because of their relation to allergies (*38*). Therefore, the physicochemical evaluation was sufficient to assess the improvement of meat matrix properties by soy protein isolate addition, but further analysis would have not been relevant.

#### Microbiological validation

The obtained value of *F*_0_ was capable of inactivating all bacteria present in the beef samples. The results for microbiology assays showed microbial counts lower than 10 CFU/g of faecal coliforms, *Clostridium, Staphylococcus*, and the absence of *Salmonella*. Retort processing in retort pouches reduces undesirable microorganisms (*39*), as reported by Rajan *et al.* (*39*) who observed that Chettinad chicken with an *F*_0_ of 5.2 min did not have any *E. coli, Salmonella* spp*., Clostridium* spp*., Staphylococci* spp, yeast and mould during 180 days of storage.

#### Syneresis and *a*_w_

During the 180 days of storage, there was no significant difference (p>0.05) in syneresis or *a*_w_, regardless of the treatment or control (data not shown). Syneresis varied around 35-45%; samples treated with collagen had overall mean of 40.56%, with modified starch 38.46%, and control samples 40.49%. The values of *a*_w_ in samples treated with collagen and modified starch were between 0.96-0.97, whereas control had an *a*_w_ of 0.96.

Cooking, as well as the presence of salt, influences the amount of water bound in the meat proteins. Cooking tends to reduce the *a*_w_, as reported by Cheon *et al.* (*5*), where sterilized meatballs at temperatures between 117 and 125 °C had an *a*_w_ of 0.96-0.99. In contrast, NaCl was able to contribute to the water retention, fat-binding properties, flavour and texture. Moreover, NaCl has a preservative effect due to its ability to decrease the water activity (*26*).

Even though the addition of hydrocolloids may retain more water in the beef samples, the results of syneresis and *a*_w_ rely on the matrix hydration. Since the meat cubes remained inside the pouches with the water lost during sterilization, the matrix was continuously hydrated, resulting in no difference in the presence of hydrocolloids. Therefore, processing (sterilizing) and salting had more influence on the syneresis and *a*_w_ than the hydrocolloid itself. However, the hydrocolloid may have more influence if the product is not immersed in the solution.

Overall, for shelf life, there was no difference in the values of *a*_w_, syneresis and lipid oxidation among all three evaluated treatments (control, collagen and modified starch). Therefore, the best treatment cannot be ranked based on a shelf life assessment.

#### Lipid oxidation

Sterilization temperatures reduce the lipid profile and composition of the matrix, depending on the heat and the imposed processing time (*39*, *40*). High temperatures favour hydrolysis, reduce the energy activation for lipid oxidation, and decompose the pre-formed hydroperoxides into free radicals, which stimulate lipid oxidation and production of off-flavours (*41*), concentration of which can increase throughout storage (*40*).

**Fig. 1:**
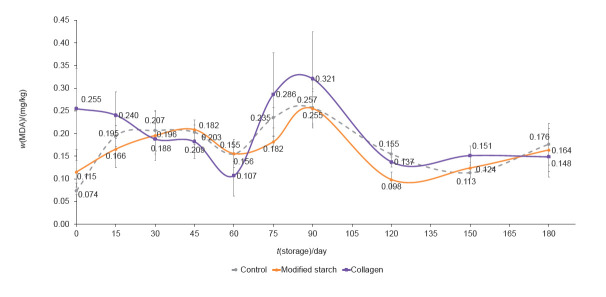
Influence of the addition of modified starch (MS) or collagen (COL) on lipid oxidation expressed as malondialdehyde (MDA) during storage of the retorted beef. CTRL=control. Data represent mean value±S.D., *N*=6

However, sterilized meat products tend to have low lipid oxidation values, as seen in the present and prior studies (*42*, *43*); lipid oxidation values expressed as malondialdehyde (MDA) ranged between 0.1 and 0.3 mgkg of product (Fig. 1). Rajkumar *et al.* (*42*) described an increase in the oxidation values (as MDA) from 0.40 to 0.97 mg/kg of the product when meat lamb was sterilized at 121.1 °C with an *F*_0_ of 12.1 min. Also, Muhlisin *et al.* (*43*) evaluated Chuncheon dakgalbi, a Korean chicken dish, sterilized at 110 °C for 30 min, and observed that product values expressed as thiobarbituric acid reactive substances (TBARS) were 1.65-2.59 mg/kg and after three weeks increased to 3.64 mg/kg.

The low oxidation values are associated with the lower effect of specific enzymes, as described by Nolletand Toldra (*44*) who observed that lipolysis could be driven by a series of enzymes, namely lipases, esterases and phospholipases, which can cleave the ester bond amid fatty acids and glycerol, resulting in the formation of free fatty acids. The extent and type of the process, such as dry-curing, ripening and fermenting can alter the extent of lipolysis and oxidation (*44*). Therefore, it can be proposed that the high sterilization temperatures are able to inactivate these enzymes, thus reducing or eliminating their functional properties.

Also, a shorter contact with oxygen favours the decrease in the TBARS values (*39*). The sterilized beef samples were vacuum packed in retort pouches composed of several layers to endure sterilization, which reduces oxygen permeability. Rajan *et al*. (*39*) observed a linear increase in lipid oxidation in retorted Chettinad chicken, which might be due to the residual oxygen as the pouches were not vacuum sealed. In addition, Güntensperger and Escher (*45*) observed a reduction of up to 80% in the sterilized ostrich meat (*F*_0_=8-9 min) when samples were stored under vacuum and modified atmosphere.

Lastly, hydrocolloids may affect oxidation, as reported by Milani and Maleki (*46*), who stated that hydrocolloids in a gel form could retard the moisture loss during a short-term storage, acting as a sacrificing agent rather than a barrier to moisture transmission. In some cases an inverse relationship between water vapour and oxygen permeability was observed, indicating that such films could provide an effective protection against lipid oxidation.

#### Texture profile, shear force and sensory properties of treates meat samples

Texture, shear force and sensory tests were made for the samples treated with collagen at 5.0% NaCl. Since collagen treatment gave the highest yield and *Y*_total_, it is optimal choice to obtain the required physicochemical properties of meat required for the industry. The presence of collagen resulted in increased hardness and shear force compared to the control (Table 4). An increase in hardness and shear force values when using collagen depends on the temperature and collagen interaction with the matrix. Damodaran *et al.* (*47*) showed that collagen tended to form protein-protein bonds, which were stronger than the bonds between meat proteins.

**Table 4:** Texture profile analysis and shear force of beef (*vastus lateralis*) cubes with 1.0% hydrocolloid and 5.0% sodium chloride

Treatment	Hardness/N	Cohesiveness	Springness/mm	Chewiness/J	Peak shear force/N
Control	(21.5±6.5)^a^	(0.25±0,02)ª	(6.6±1.2)ª	(20.2±2.6)ª	(21.2±4.2)^a^
Collagen	(60.3±18.8)^b^	(0.41±0.02)^b^	(9.9±1.4)^b^	(211.6±36.9)^b^	(38.4±3.7)^b^

Data represent mean value±S.D., *N*=4. Mean values in the same column with different letters in superscript are significantly different (p<0.05) according to the Tukey’s test

In addition, temperatures above 60 °C shorten the collagen fibres, which reduces the volume of muscular fibres and consequently, its hardness (*48*). In the range 80-120 °C, gelatinization of the soluble collagen led to a reduced fibre diameter and sarcomere length (*3*). In contrast, control samples were softer, with values of hardness and peak shear force of 21.5 and 21.2 N, respectively. Palka (*3*) had similar values, 21.34 N, for hardness of the sterilized beef without additives. Lower hardness values were also observed in sterilized short rib patties, with the lowest temperatures being 121-125 °C (*10*).

The control samples received the highest number of preferences, with a statistically significant higher score (p>0.05), with 46 preferences out of 74. This may be due to lower hardness, shear force and chewiness values than for the samples treated with collagen (Table 4). Nonetheless, the panellists did not perceive the presence of collagen in the triangle test, whereas the number of incorrect answers was 48 out of the total 74 answers (p>0.05). Sensory analyses published by Prestes *et al*. (*49*) had no statistical differences when sausages with 1 and 4% collagen were compared to the control, showing that the addition of collagen made no difference to the panellists. Daigle *et al*. (*50*) stated that the addition of 0.3% carrageenan, 1.5% soy protein isolate or 1.5% collagen did not affect consumer acceptance of the pale, soft and exudative-like (PSE-like) turkey breast.

## CONCLUSIONS

Retort provides a convenient, safe and ready-to-eat meat products. Nonetheless, high temperatures lower water holding capacity, influencing the texture, yield and water retention. Since there is a lack of studies that bypass the harmful effects of this technology, the present work studied the benefits of water-binding properties of hydrocolloids combined with salt on sterilized meat products. Results showed that even under the harsh conditions of sterilization, lipid oxidation (expressed as malondialdehyde) remained at low levels of 0.1-0.3 mg/kg during 180 days of storage. The addition of salt combined with hydrocolloids resulted in a higher yield, better water-holding capacity and minimized cooking loss due to a higher water retention. Treatment with 1% collagen and 5.0% NaCl gave the highest yield (17.7%), whereas control had 11.6%. The addition of hydrocolloids did not influence water activity or syneresis levels of the product. However, samples treated with collagen had the best physicochemical results overall but also a harder and firmer meat than the control samples. Although sensory analysis showed a preference for control over the samples treated with collagen, panellists did not perceive the presence of collagen in the product. Retort technology can be improved by the addition of hydrocolloids combined with salt for the improvement of quality of the final product.
